# The Role of Endoscopic Ultrasound in Ampullary Lesion Management

**DOI:** 10.3390/diagnostics14171855

**Published:** 2024-08-25

**Authors:** Caterina Stornello, Chiara Cristofori, Davide Checchin, Maria Grazia de Palo, Sabina Grillo, Giulia Peserico, Dario Quintini, Mario Gruppo, Ottavia De Simoni, Alberto Fantin

**Affiliations:** 1 Gastroenterology Unit, Veneto Institute of Oncology IOV-IRCCS, 35100 Padua, Italy; chiara.cristofori@iov.veneto.it (C.C.);; 2 Gastroenterology Unit, Dell’Angelo Hospital, 30174 Venice, Italy; davide.checchin@aulss3.veneto.it; 3 Unit of Surgical Oncology of Digestive Tract, Veneto Institute of Oncology IOV-IRCCS, 35100 Padua, Italyottavia.desimoni@iov.veneto.it (O.D.S.)

**Keywords:** endoscopic ultrasound, ampullary tumor, familial adenomatous polyposis

## Abstract

Ampullary lesions, neoplasms originating in the papilla of Vater, represent a rare yet clinically significant group of tumors with diverse etiologies and management challenges. This comprehensive review aims to elucidate the pivotal role of endoscopic ultrasound (EUS) in the diagnosis, staging, and management of ampullary lesions. This review begins by providing an overview of ampullary lesions, their epidemiology, and associated risk factors. We delve into their clinical presentation, emphasizing the importance of early and accurate diagnosis. Furthermore, we explore the limitations of traditional diagnostic modalities and highlight the growing relevance of EUS in ampullary lesion evaluation. We discuss the superior spatial resolution of EUS in comparison with other imaging methods, and we present an in-depth analysis of EUS-guided sampling and its pivotal role in obtaining histological samples for accurate diagnosis. In addition to diagnosis, we examine the indispensable role of EUS in ampullary lesion staging and its clinical implications. Furthermore, we discuss the potential of EUS in the surveillance and follow-up of ampullary lesions, ensuring timely detection of recurrence and monitoring treatment response in sporadic cases and in the context of familial syndromes, such as familial adenomatous polyposis (FAP). In conclusion, this review underscores the indispensable role of endoscopic ultrasound in the multifaceted approach to ampullary lesion evaluation. EUS not only enhances diagnostic accuracy but also informs treatment decisions and minimally invasive therapeutic interventions. As our understanding of ampullary lesions continues to evolve, EUS remains an invaluable tool for the improvement of patient outcomes and quality of life.

## 1. Introduction

Ampullary lesions, neoplasms originating in the region of the ampulla of Vater, represent a rare yet clinically significant distinct clinicopathologic entity of tumors that pose unique diagnostic and management challenges. Their incidence is about 1 per 100,000 each year, and although they account for a small percentage of gastrointestinal malignancies (0.6–0.8%), they are the most common neoplasm in the small bowel [1]. Tumors of the ampulla include adenomas, adenocarcinomas in an adenoma–carcinoma sequence, and neuroendocrine tumors [2]. Given their strategic location at the confluence of the distal common bile duct and the pancreatic duct, ampullary lesions can manifest with a spectrum of symptoms, such as obstructive jaundice, gastrointestinal bleeding, anemia, acute pancreatitis, anorexia, weight loss, and abdominal discomfort. Their diagnosis may also be occasional in asymptomatic patients, thus leading to increased incidence in the last twenty years, probably due to the wider availability of endoscopy and imaging [3,4].

The early and accurate diagnosis of ampullary lesions is paramount to guide appropriate management strategies with the aim of improving patient outcomes since the potential for curative resection is diagnosed at an early, localized stage. In fact, when a prompt diagnosis of an ampullary tumor is made, correct staging is mandatory in order to adequately address the patient with an endoscopic or surgical approach [5,6].

This review seeks to elucidate the multifaceted role of EUS in the study of ampullary lesions by synthesizing existing knowledge and recent advancements in the field.

## 2. Diagnosis

As anticipated, the diagnosis of ampullary tumors is rare, but thanks to better-quality endoscopy, their recognition is increasing. Traditionally, the diagnosis and characterization of ampullary lesions have long relied on traditional diagnostic modalities, including computed tomography (CT) and magnetic resonance imaging (MRI), esofagogastroduodenoscopy (EGDS), and histopathological assessment [7]. In particular, the latest guidelines recommend accurately evaluating the region of the papilla major during each EGDS and undertaking biopsies in cases of suspicion [1]. Also, the ESGE guidelines recommend the study of papilla with a cap-assisted gastroscope [1]. When a histological diagnosis of ampullary adenoma is made, management changes are made according to the grade of dysplasia and, of course, locoregional and systemic staging. Although CT and MRI scans are useful in identifying distant metastases and are employed for the initial evaluation of patients with suspected ampullary lesions [8], these imaging modalities face several challenges: suboptimal resolution due to the small area considered, difficulty in distinguishing benign and malignant lesions, and an inability to assess the depth of invasion. The recognition of these limitations has led to the growing prominence of EUS as a pivotal tool in the study of ampullary lesions [9,10]. 

EUS, an endoscopic imaging technique with high spatial resolution and real-time capabilities, offers unparalleled advantages in visualizing the papilla and adjacent structures and might also play a crucial role in ampullary tumor locoregional staging, aiding in the assessment of tumor size, the depth of invasion, and lymph node involvement.

Its outstanding precision can also help in staging tumors according to the TNM classification; in particular, the eighth edition of AJCC states the following: Tis: carcinoma in situ; T1: tumor limited to the ampulla of Vater and the sphincter of Oddi, or tumor invades beyond the sphincter of Oddi (perisphincteric invasion) or into the duodenal submucosa; T1a: tumor limited to the ampulla of Vater or sphincter of Oddi; T1b: tumor invades beyond the sphincter of Oddi (perisphincteric invasion) or into the duodenal submucosa; T2: tumor invades into the muscularis propria of the duodenum; T3: tumor directly invades into the pancreas (up to 0.5 cm) or tumor extends more than 0.5 cm into the pancreas or extends into peripancreatic or periduodenal tissue or duodenal serosa without involvement of the celiac axis or superior mesenteric artery; T3a: tumor directly invades the pancreas (up to 0.5 cm); T3b: tumor extends more than 0.5 cm into the pancreas or extends into peripancreatic tissue or periduodenal tissue or duodenal serosa without involvement of the celiac axis or superior mesenteric artery; and T4: tumor involves the celiac axis, superior mesenteric artery or common hepatic artery, irrespective of size [11].

Few studies have investigated the differences between radial and linear EUS. Some years ago, a prospective, randomized trial included 200 patients undergoing EUS for pancreaticobiliary diseases. In total, 99 patients were randomized to the radial scope and 101 patients to the linear scope. The principal aim of the trial was the basic imaging capability to evaluate the entire pancreaticobiliary region by assessing eleven areas and assigning one of three scores each. Overall, the non-inferiority of the imaging capability of the linear scope compared to that of the radial scope was demonstrated. Although linear scope was superior in the depiction of some anatomical regions, such as the pancreatic head, neck, and the area from the hepatic portal vein to the superior bile duct, the delineation of the major duodenal papilla and gallbladder were better evaluated through radial scope [12].

However, more recently, a very huge population was retrospectively analyzed to specifically demonstrate the rate of complete visualization of the pancreatobiliary area with radial or linear scope.

In total, 1660 radial EUSs and 1984 linear EUSs were recruited. The success rates of visualization of the pancreatobiliary junction were significantly better for linear scope than the radial one (89.5% vs. 80%, respectively; *p* < 0.0001) [13].

A few years ago, a meta-analysis was conducted to evaluate the performance of EUS. It included 422 patients from 14 studies. In T1 tumors, the pooled sensitivity and specificity of EUS were 77% (95% CI 69–83%) and 78% (95% CI 72–84%), respectively. Pooled sensitivity for T4 tumors was 84% (95% CI 73–92%), and specificity was 74% (95% CI 63–83%). It was also demonstrated that the pooled sensitivity and specificity of morphological criteria for lymph node involvement were 70% (95% CI 62–77%) and 74% (95% CI 67–80%), respectively [14].

Also, a number of comparative studies were conducted to better assess the role of EUS. In a paper based on 23 patients with ampullary cancer, EUS had a high tumor detection rate (96%) and was better than abdominal ultrasonography (US) and CT scans. Using EUS, it was also possible to stage the magnitude of these tumors according to the involvement of the duodenal or CBD walls, the invasion of the pancreas and portal vein, and the involvement of regional lymph nodes: the accuracy rates of cancer extent obtained by EUS were 78% [15].

Similarly, a prospective evaluation on 27 patients affected by ampullary cancer and staged with a CT scan and EUS concluded that CT findings do not improve the test performance characteristics of EUS. In effect, in this study, the strength of tumor (kappa 0.51 vs. 0.11) and nodal (kappa 0.59 vs. 0.05) agreement with final pathology was statistically and significantly higher for EUS than for CT (*p* < 0.05): EUS was more sensitive and specific than CT for both tumor and nodal staging [16].

Nodal evaluation is paramount in staging ampullary neoplasm, which is one of the findings that drive treatment decisions. EUS efficacy in this sense was investigated in a cohort of 120 patients undergoing resection for ampullary adenoma. In those receiving both EUS nodal staging and surgery, the overall accuracy of EUS in regional nodal staging was 75.0%. In detail, the sensitivity and specificity for N0 disease were 87.5% (95% CI 64.5–100.0%) and 62.5% (95% CI 29.0–96.1%), respectively; for N1 disease, they were 62.5% (95% CI 29.0–96.1%) and 87.5% (95% CI 64.5–100.0%), respectively [17]. In addition, when compared with other imaging modalities, such as CT and MRI, EUS is already known to have similar accuracy in detecting malignant regional lymph nodes before surgery (EUS 68%, CT 59%, MRI 77%) in both adenocarcinomas and adenomas [18].

A retrospective study conducted on seventy-four patients with suspicion of a periampullary tumor also demonstrated the fundamental role of EUS. Thirty-six patients underwent surgery, and the local staging accuracy of the imaging modalities was compared for them. EUS was the most sensitive modality in the depiction (97% vs. 39% for CT; *p* < 0.001) and T classification (72% vs. 22% for CT) of periampullary tumors. It was also explored if the accuracy of the diagnosis was affected by the presence of a biliary stent, resulting in a minimal but not significant variation in the results [19].

As far as MRI is concerned, although it is historically regarded as the most reliable noninvasive diagnostic imaging examination for studying the biliary tree and pancreatobiliary lesion, it might not be perfectly accurate for the evaluation of the papilla of Vater. In fact, it is a possible blind spot for MRI because of its small size and the tapering of the intramural ducts that may contain little fluid [20]. A linear EUS and MRI were then compared in a cohort of 24 patients affected by ampullary tumor. During EUS, multiple biopsies were taken by endoscopic forceps, and the agreement between endoscopic and histological results was very good (κ = 0.81). In 25% of cases, MRI examination was negative; in 71% cases, it only showed indirect signs such as the dilation of intrahepatic and extrahepatic bile ducts, and in 8% of cases, it showed an actual space occupying the lesion of the ampulla. On the contrary, EUS detected ampullary tumors in all patients (*p* < 0.03), proving to be extremely accurate, letting it, at the same time, obtain direct endoscopic visualization of the major duodenal papilla, taking biopsies, and depicting the layered structures of the periampullary area [21,22,23].

Furthermore, differing from CT and MRI, EUS images can be enhanced using some dynamic tools. In fact, inflating the balloon and adding water in the duodenum through the working channel of the echoendoscope allows the underwater evaluation of the papilla so that it is free of bubbles. A very clear evaluation of the ampulla and of all the wall layers is made, making T staging extremely precise [24]. Recently, a new technique explored the opportunity of a better visualization of the ampullary region compared with water. Sato and colleagues compared the images obtained both with the endoluminal instillation of water (firstly), and with the endoluminal injection of gel (secondly, after complete aspiration of the water). The primary outcome was the number of images illustrating the pancreatic and biliary ducts penetrating the duodenal muscolaris propria in each technique. The secondary outcome was for the gel immersion technique’s safety and impact on duodenal lumen distension.

Ten patients’ exams were analyzed. The primary outcome was significantly higher for the gel-immersion technique, demonstrating a better exploration of the merging of the ducts in the duodenum. Moreover, no adverse events were registered, and the subanalysis revealed similar results both with convex and radial echoendoscopes [25]. 

Similarly, in a small cohort of 12 patients affected by papillary carcinomas who underwent surgery after EUS using the gel immersion technique, the diagnostic accuracy of biliary spread, pancreatic intraductal spread, invasion into the duodenum’s muscularis propria, and pancreatic invasion were 83%, 100%, 83%, and 92%, respectively [26].

EUS can also benefit from some ancillary techniques, such as contrast harmonic EUS (CH-EUS) and elastography. However, in the literature, there are only a few dated studies evaluating the use of CH-EUS in the study of ampullary lesions and even fewer concern the use of elastography. It is well known that the detection of a hypoenhancing and inhomogeneous mass in CH-EUS accurately identifies patients with malignant neoplasia. CH-EUS, moreover, increases the detection of malignant lesions in difficult cases (such as patients with chronic pancreatitis or biliary stents) and helps to guide EUS-FNAB. An old study from 2010 [27] aimed at demonstrating how CH-EUS increases diagnostic accuracy in the pre-operative staging of tumors of the biliary-pancreatic tract confirmed that CH-EUS helped to more clearly investigate the depth of invasion of the ampullary neoplasm. 

## 3. Sampling

Histopathological examination remains the gold standard for confirming the nature of ampullary lesions. However, it has its own set of limitations:

Sampling errors: biopsies taken during endoscopy may not adequately represent the entire lesion, leading to sampling errors and potential misdiagnosis.

Inflammatory changes: ampullary inflammation or tissue reactions to obstruction can sometimes mimic malignancy, leading to misinterpretation.

Limited information on depth of invasion: standard biopsy specimens may not provide sufficient information about the depth of tumor invasion into the ampullary wall.

Given these limitations, there is a pressing need for more accurate and reliable diagnostic approaches for ampullary lesions, particularly those that can provide detailed information about lesion characteristics, including their size, extent, and malignancy status.

Endoscopic biopsy for histological examination with routine hematoxylin and eosin-stained sections is mandatory in the diagnosis of ampullary tumors. The accuracy of a standard forceps biopsy has been reported with wide variation, ranging from 38.3% to 85%, showing a consistent risk of inadequate follow-up treatment [1].

Biopsy can be safely performed with standard forceps through the linear echo endoscope channel, similar to the method used with lateral vision endoscopes or with dedicated fine needle aspiration (FNA) or fine needle biopsy (FNB). The use of needles seems to be safe and accurate (88%), with a sensitivity and specificity of 82.4% and 100%, respectively [8,28]. These data come from studies performed with FNA needles; data from first and second generation FNB needles are scarce. Although these data would be of great interest, considering that in clinical practice, FNB is widely performed, even more than FNA, this is also due to the absence of rapid-on-site-evaluations in a lot of centers.

In the case of a bulging papilla, several reports suggest that endoscopic biopsies should be performed after sphincterotomy, but data are conflicting, for example, when considering the risk that post-sphincterotomy changes in histology can mimic cytoarchitectural atypia [29,30]. It was even reported that sphincterotomy can reduce diagnostic accuracy by 25%, although it has been suggested that repeated further sampling ten days after can increase the performance of this method [31].

Therefore, FNA can be safely used as an alternative method to confirm the diagnosis and to avoid the complications linked with sphincterotomy to expose an underlying tumor [32,33]. FNA cytology has a major limitation, which is its inability to fully characterize the spectrum of cytopathological changes from reactive atypia to high-grade dysplasia and invasive adenocarcinoma, which requires histological analysis not provided by routine cytologic samples. To sample suspicious malignant pancreatic tumors, core biopsy needles are being increasingly utilized, but data on their role in ampullary cancers are less clear in the literature [34]. In addition, EUS-guided FNA might also have a role in sampling tissue from periduodenal adenopathy since it is highly accurate, so the use of this technique might increase the diagnostic accuracy of preoperative EUS, although supportive data are limited. Also, ESGE guidelines suggest considering EUS FNA/B when an obstructive ampullary tumor is suspected that has initial negative histopathology [1].

## 4. Staging

It is nowadays recognized that, when feasible, the preferable treatment for ampullary adenomas is endoscopic resection over surgery because of a lower morbidity rate with similar rates of mortality, margin-positive excision, and reinterventions [35]. Intraductal growth has been shown to make endoscopic papillectomy more technically challenging [36]. However, although some authors consider endoscopic treatment in cases of intraductal growth limited to 1 cm [37], no consensus has been reached regarding whether or not the tumor should extend into the biliary and pancreatic ducts, and currently, both ESGE and Japanese guidelines recommend endoscopic treatment in cases without intraductal growth [1,38].

In this sense, accurate staging must give details about intraductal involvement, this being a cut-off criterion for the endoscopic versus surgical approach.

A recent meta-analysis that included 21 studies explored the diagnostic accuracy of EUS for detecting tumor depth (T-staging). The pooled sensitivity and specificity of EUS were 0.89 and 0.87 for T1, 0.76 and 0.91 for T2, 0.81 and 0.94 for T3, and 0.72 and 0.98 for T4, respectively [39]. 

Among intraductal biliopancreatic imaging techniques, the role of intraductal ultrasonography (IDUS) has also been investigated. In a single-center, prospective, and histopathologically controlled study of forty patients, EUS with a radial echoendoscope and IDUS were performed. Although a small percentage of patients were over-staged, in 10 patients who underwent endoscopic papillectomy, the accuracy of IDUS in T staging was superior to that of EUS (100% vs. 80%). Tumor extension into the biliary duct and the pancreatic duct was correctly assessed in 88% and 90% of patients by EUS and in 90% and 90% by IDUS, respectively [40]. More recently, in a study of forty-one patients, extension into the bile and pancreatic ducts was successfully evaluated in all patients, and extension into the bile duct was evaluated with an accuracy of 88%, not differing from EUS accuracy [41].

Interesting results came from a wider cohort of 234 patients with indeterminate bile duct strictures, in which multiple modalities were compared, including IDUS. Final diagnoses were both malignant and benign, and IDUS and endoscopic retrograde cholangio-pancreatography were superior to EUS and CT in providing accurate diagnoses of bile duct strictures of uncertain etiology [42].

In summary, IDUS for T-staging in ampullary tumors has been reported to have overall accuracies of between 78% and 90.2%. Most reports suggest that diagnostic yields of IDUS are just slightly higher than or comparable to those of EUS. Notably, IDUS could be useful to guide direct tissue acquisition in biopsy or brush cytology. However, this method implicates a considerable risk of pancreatitis, and in clinical practice, costs and risks must be balanced. For these reasons, ESGE guidelines do not recommend the routine use of IDUS in staging ampullary neoplasm, but they suggest it can be useful in selected patients [1].

## 5. Role of EUS in Evaluating Ampullary Lesions in Familial Adenomatous Polyposis (FAP)

The majority of benign or malignant ampullary tumors are sporadic and involve major papilla. Nevertheless, when the diagnosis is made at a younger age, a genetic predisposition should be suspected. FAP syndrome represents the strongest hereditary predisposition with a 120-fold increased relative risk compared to the general population [43]. Other rarer predisposing genetic syndromes have been described, such as neurofibromatosis type I (not only for somatostatinomas but also for carcinoma) or Muir–Torre syndrome [44].

FAP syndrome is a complex disease, which includes FAP, attenuated FAP, and MUTYH-associated polyposis (MAP), and this is a rare genetic disorder characterized by the development of numbers of adenomatous polyps throughout the colon and rectum [45].

These patients are at significant risk for colorectal cancer and incur additional risks for extracolonic malignancies. The primary concern in FAP is colorectal cancer, but subjects with FAP are also at an increased risk of developing ampullary adenomas. Given the predilection for malignancy to occur at and near the papilla in patients with FAP and attenuated FAP, this area must be carefully evaluated. Adenomas are found at the ampulla in up to 72% of stage IV FAP patients [46]. Interestingly, adenomatous changes can also be found in macroscopically normal-appearing papilla in 29% to 54% of individuals undergoing random biopsy sampling [47,48]. There are also reports of developing ampullary cancer in patients with a normal papilla upon index screening.

In MAP, the prevalence of duodenal adenomas is lower than in patients with FAP, with 17–34% at a median age of 50 years. Only 6% of these patients with MAP developed ampullary disease (both adenomas and cancers) [49,50]. Duodenal polyposis occurs later in life and has a slower progression than in individuals with FAP, so duodenal surveillance may commence at an older age.

The two main histological subsets of the precursor lesions of benign or malignant ampullary tumors are from intestinal-type mucosa as well as from pancreatic duct-type ampullary mucosa [44]. The intestinal type evolves through a well-known adenoma–carcinoma sequence. The pancreaticobiliary type evolves from precursor pancreatic duct intraepithelial neoplasia. The staging of the lesion has to be based on the latest TNM classification [51].

In this special context, the role of EUS becomes even more critical. In the literature, endoscopic ultrasonography for the pretherapeutic staging of ampullary tumors has focused mainly on advanced ampullary cancers. In fact, ESGE suggests that endoscopic ultrasonography should not be routinely performed in the pretherapeutic evaluation of ampullary adenomas in individuals with familial adenomatous polyposis/MUTYH-associated polyposis [52]. It may be considered for the assessment of large or suspicious papillae to help exclude invasive growth, for the staging of an ampullary tumor, or during follow-up. Furthermore, ESGE also suggests the use of IDUS in the evaluation and staging of selected patients with an overall accuracy between 78% and 90.2% [41,53,54,55].

EUS, in fact, provides the precise localization of ampullary lesions within the duodenal wall. It aids in characterizing the size, morphology, and characteristics of these lesions. The accuracy of EUS in confirming that the T stage is higher than T1 is around 90%, and its ability to predict the intraductal extension of the tumor is accurate (in studies using surgical resection as the reference standard) [34]. This information is essential for determining the nature of the lesion and guiding treatment decisions. 

As previously pointed out, a comparison of the preoperative staging of ampullary tumors showed the comparable accuracy of EUS and IDUS. On the other hand, over-staging at EUS/IDUS occurred in 25–40% of cases of benign adenoma or early cancers. Therefore, EUS and IDUS offer limitations in their pretherapeutic evaluation of ampullary tumors, with the over-staging of benign lesions at early stages.

However, in symptomatic patients presenting with jaundice, the sensitivity of EUS in the diagnosis of an ampullary tumor remains high, but it is lower in asymptomatic cases. For example, it is not uncommon for patients with FAP to harbor ampullary tumors without EUS abnormalities. This emphasizes the fact that, despite the high sensitivity of EUS for the diagnosis of ampullary tumors, its negative predictive value remains limited, and sometimes, only a mucosal biopsy can faithfully confirm the diagnosis.

Furthermore, if an ampullary lesion is identified and deemed high risk, EUS can guide minimally invasive interventions, such as EUS-guided FNA/B, or address endoscopic papillectomy. These procedures can provide histological confirmation, and thereafter, endoscopic papillectomy may be curative, depending on the stage of the lesion. Performance seems to be safe and accurate, with sensitivity at 82%, specificity at 100%, and accuracy at 88.8% [33].

In summary, EUS can be helpful in the diagnosis of ampullary tumors, especially lesions with intramural spread and negative mucosal biopsies. Its role in guiding the strategy of management of ampullary cancer lies in its ability to accurately stage advanced tumors where surgical resection can be performed. For benign and early cancers of the ampulla, accurate staging could be achieved using a combination of endoscopic, EUS, IDUS, and cross-sectional imaging.

## 6. Conclusions

EUS has emerged as an indispensable tool in the comprehensive study of ampullary lesions, offering a multitude of advantages that significantly enhance diagnostic accuracy, staging capabilities, and patient care. Through its high spatial resolution, real-time imaging, and EUS-guided interventions, EUS has reshaped our approach to ampullary lesion evaluation.

Incorporating EUS into the diagnostic algorithm for ampullary lesions has revolutionized our approach to these neoplasms, enhancing diagnostic precision, staging accuracy, and overall patient care. Its pivotal role in addressing the limitations of traditional diagnostic modalities underscores the importance of EUS in the evaluation and management of ampullary lesions. 

The ability of EUS to provide detailed insights into ampullary lesions, including their size, depth of invasion, lymph node involvement, and impact on adjacent structures, empowers clinicians with crucial staging information. This information, in turn, guides treatment decisions, ensuring that patients receive personalized care tailored to their specific disease stage.

In special situations like FAP, where the risk of ampullary adenomas is heightened, EUS takes on an even more critical role. It enables the precise localization, characterization, and proactive surveillance of ampullary lesions, reducing the risk of malignancy and optimizing outcomes for individuals with FAP.

As we continue to advance in the field of gastroenterology, EUS stands as a testament to innovation and progress. Its unique capabilities have not only improved diagnostic accuracy but have also expanded the possibilities for minimally invasive interventions, thereby enhancing the quality of life for patients with ampullary lesions.

In conclusion, EUS has firmly established itself as an indispensable asset in the study of ampullary lesions. Its role in detection, accurate staging, and tailored interventions cannot be overstated. Through EUS, we gain the power to decipher the complexities of ampullary lesions, unlocking the potential for timely intervention and improved patient outcomes.
